# Exploring the nasopharyngeal microbiota composition in infants with whooping cough: A test-negative case-control study

**DOI:** 10.1371/journal.pone.0259318

**Published:** 2021-10-29

**Authors:** Muntsa Rocafort, Desiree Henares, Pedro Brotons, Irene Barrabeig, Cristian Launes, Lore Merdrignac, Marta Valenciano, Angela Domínguez, Pere Godoy, Carmen Muñoz-Almagro

**Affiliations:** 1 Institut de Recerca Sant Joan de Déu, Sant Joan de Deu Hospital, Barcelona, Spain; 2 CIBER of Epidemiology and Public Health (CIBERESP), Instituto de Salud Carlos III, Madrid, Spain; 3 School of Medicine, Universitat Internacional de Catalunya, Barcelona, Spain; 4 Department of Health, Public Health Agency of Catalonia, Barcelona, Spain; 5 Epidemiology Department, Epiconcept, Paris, France; 6 Department of Medicine, University of Barcelona, Barcelona, Spain; Universidad Nacional de la Plata, ARGENTINA

## Abstract

**Purpose:**

The purpose of this study was to characterize the nasopharyngeal microbiota of infants with possible and confirmed pertussis compared to healthy controls.

**Methods:**

This prospective study included all infants <1 year with microbiologically confirmed diagnosis of pertussis attended at a University Hospital over a 12-month period. For each confirmed case, up to 2 consecutive patients within the same age range and meeting the clinical case definition of pertussis but testing PCR-negative were included as possible cases. A third group of asymptomatic infants (healthy controls) were also included. Nasopharyngeal microbiota was characterized by sequencing the V3-V4 region of the 16S rRNA gene. Common respiratory DNA/RNA viral co-infection was tested by multiplex PCR.

**Results:**

Twelve confirmed cases, 21 possible cases and 9 healthy controls were included. Confirmed whooping cough was primarily driven by detection of *Bordetella* with no other major changes on nasopharyngeal microbiota. Possible cases had limited abundance or absence of *Bordetella* and a distinctive microbiota with lower bacterial richness and diversity and higher rates of viral co-infection than both confirmed cases and healthy controls. *Bordetella* reads determined by 16S rRNA gene sequencing were found in all 12 confirmed cases (100%), 3 out of the 21 possible cases (14.3%) but in any healthy control.

**Conclusion:**

This study supports the usefulness of 16S rRNA gene sequencing for improved sensitivity on pertussis diagnosis compared to real-time PCR and to understand other microbial changes occurring in the nasopharynx in children <1 year old with suspected whooping cough compared to healthy controls.

## Introduction

Whooping cough, also known as pertussis, is a highly transmissible acute respiratory infection mainly caused by the bacterium *Bordetella pertussis*. Other *Bordetella* species such as *B*. *parapertussis*, *B*. *holmesii* and *B*. *bronchiseptica* have also been associated with pertussis-like illness [1]. Despite initial symptomatology resembles that of a common cold, whooping-cough characteristic manifestations usually appear after 10 to 15 days [2]. Pertussis affects all population ages, but while adolescents and adults usually develop mild symptoms, the disease can be especially life-threatening in unvaccinated and incompletely vaccinated young infants (<1 year) [3]. Particularly, most severe complications (including pneumonia, seizures and encephalopathy) and deaths occur in infants aged <6 months and half of infants younger than 1 year require hospitalization [4, 5].

Since 1965, vaccination against *B*. *pertussis* has been covered by the Spanish public health system and pertussis has been a mandatory notifiable disease since 1981. After the introduction of vaccine programs in the 60s, the incidence of *B*. *pertussis* decreased significantly. However, in recent years, before the Covid-19 pandemic, a resurgence of whooping cough was observed in Catalonia, Spain [6, 7], similarly to other European countries [8].

Clinical case definition of pertussis established by the European Centre for Disease Prevention and Control (ECDC) is based on a variety of symptoms compatible with *B*. *pertussis* infection [9]. Nonetheless, its sensitivity and specificity are low due to the wide clinical spectrum of whooping-cough. Confirmed cases are considered if confirmation is obtained by microbiological laboratory tests, otherwise, they are considered as possible cases. Bacterial culture and nucleic acid amplification tests (NAATs) against specific *Bordetella* genes are the recommended tests for microbial diagnosis, especially in young children, but both of them have limitations [10]. Bacterial culture remains the gold standard because of its high specificity, but it has limited sensitivity because of low cell survival during specimen transport and culture requirements [11, 12]. Amplification of *Bordetella*-specific genes by polymerase chain-reaction (PCR), first implemented in 1989 [13], has higher sensitivity and specificity, but PCR techniques need to be periodically updated to ensure that adaptations or changes in pertussis target genes do not compromise the results [14].

New molecular methods based on next generation sequencing (NGS) of the 16S rRNA gene have demonstrated to be useful tools to investigate low microbial-populated ecological niches [15] and to better understand the etiology and host-related factors of respiratory diseases [16]. Studies on respiratory microbiome have shown that specific microbiota profiles can be associated with an increased risk and frequency of respiratory infections, disease severity and chronic respiratory disease in childhood [17]. In addition, different authors have suggested that nasopharyngeal microbiota analysis could be useful for undirected diagnosis of bacterial infections [18, 19].

The primary objective of this study was to characterize the composition of the nasopharyngeal microbiota of infants with possible pertussis (only meeting clinical criteria) and confirmed pertussis compared to healthy controls. The secondary objective was to assess the potential of nasopharyngeal microbiota composition for accurate diagnosis of whooping cough.

## Materials and methods

### Design and study population

This was a test-negative case-control study design that prospectively included all infants younger than 1 year old attended at University Hospital Sant Joan de Déu (HSJD) in Barcelona (Spain) with case-definition clinical criteria and microbiological confirmation of whooping cough from January to December 2016. For each confirmed case, up to 2 consecutive patients within the same age range (<1 year old) and time-period and meeting the clinical case definition of pertussis but testing PCR-negative were also included as possible cases (test-negative controls). A third group of asymptomatic infants similar in age and time of recruitment to confirmed and possible cases were selected among those attending HSJD for a routine pediatrician check-up or for blood extraction before minor surgery (healthy controls). Exclusion criteria included fever and/or active antibiotic treatment at the time of study inclusion, as well as antibiotic use within the month prior to sample collection.

HSJD is a tertiary children’s hospital that manages around 20% of total hospitalizations of infants younger than 1 year in Catalonia (Spain), a region with a population of 7,462,722 people (70,185 of them infants <1 year). Pertussis vaccination schedule in 2016 included pregnant women between 27–36 gestational weeks, infants at their 2nd, 4th, and 11th months of life and children of 6 years of age.

### Clinical definition of whooping cough

All symptomatic participants were clinically diagnosed as suspected of whooping cough, tested by real-time PCR, and prescribed azithromycin antimicrobial treatment until availability of PCR results. Criteria used for clinical diagnosis of pertussis followed those defined by ECDC [9] and based on results from a *Bordetella* specific real-time PCR as the laboratory criterion, we defined possible cases as any infant meeting the clinical criteria and confirmed cases as any infant meeting the clinical and laboratory criteria.

### Data collection

Only participants with informed consent were included in the study. Epidemiological information was collected during interviews with the families. Clinical data was retrieved from medical records. Microbiological variables obtained through laboratory analyses included *B*. *pertussis* bacterial DNA load, bacterial culture results and detection of major respiratory viruses. A description of epidemiological, clinical and microbiological variables is shown in Table 1.

**Table 1:** Epidemiological, clinical, and microbiological characteristics of participants.

		Confirmed cases (n = 12)	Possible cases (n = 21)	Healthy controls (n = 9)	P-value		
					Overall	Cases vs healthy controls	Confirmed vs possible cases
**Epidemiological characteristics**							
Gender, female		9 (75%)	12 (57.1%)	7 (77.8%)	0.351^a^	0.69^b^	0.516^b^
Median age, months (IQR)		6 (2–7.8)	5 (3–6)	6 (2–8)	0.857^c^	1^e^	0.597^e^
Siblings	No	7 (58.3%)	9 (42.9%)	6 (66.7%)	0.525^a^	0.842^a^	0.322^a^
	1		2 (16.7%)	9 (42.9%)				2 (22.2%)
	2 or more	3 (25.0%)	3 (14.3%)	1 (11.1%)			
Country of origin, Spain		12 (100%)	21 (100%)	9 (100%)	1^a^	1^a^	1^a^
Mode of delivery, C-section		3 (25%)	7 (33.3%)	3 (33.3%)	0.912^a^	1^b^	0.915^b^
Median gestational age, weeks (IQR)		40 (38–40)	39 (38–40)	40 (39–41)	0.254^c^	0.109^e^	0.699^e^
Mean weight at birth, grams (SD)		3059 ± 496	3142 ± 576	3095 ± 565	0.915^d^	0.128^f^	0.667^f^
Breastfeeding		10 (83.3%)	16 (76.2%)	7 (77.8%)	1^a^	1^b^	0.968^b^
Median breastfeeding duration, weeks (IQR)		10 (8–26)	13.5 (9.5–20)	16 (6–20)	0.927^c^	0.766^e^	0.798^e^
Infant Day Care	Kindergarten	0 (0%)	6 (28.6%)	1 (11.1%)	0.094^a^	0.429^a^	0.115^b^
	Home care	12 (100%)	15 (71.4%)	8 (88.8%)			
Parental active tabaco use		3 (27.3%)^3^	7 (33.3%)	6 (66.7%)	0.132^a^	0.124^b^	1^b^
Mean cigarettes/day (SD)		9 ± 8.5	10 ± 8.3	11.7 ± 11.2	0.939^d^	0.727^f^	0.899^f^
***Bordetella pertussis* vaccination**	**Mother**	**5 (41.7%)**	**20 (95.2%)**	-	-	-	**<0.01** ^**b**^
	Child^1^	7 (58.3%)	17 (81%)	8 (88.9%)	0.254^a^	0.570^**b**^	0.319^**b**^
**Clinical characteristics**							
Underlying conditions*		2 (16.7%)	1 (4.8%)	1 (11.1%)	0.531^a^	1^a^	0.538^a^
Time since symptoms onset until sample collection (days)		8 (6, 14.8)	5.5 (3, 8)	-	-	-	0.075^e^
Symptoms on admission	Cough	12 (100%)	21 (100%)	-	-	-	1^a^
	Emetic cough	3 (25%)	12 (57.1%)	-	-	-	0.155^b^
	Paroxysm	11 (91.7%)	15 (71.4%)	-	-	-	0.355^b^
	Inspiratory stridor	6 (50%)	6 (28.5%)	-	-	-	0.393^b^
	Apnoea	2 (16.7%)	4 (19%)	-	-	-	1^a^
	Cyanosis	2 (16.7%)	0 (0%)	-	-	-	0.125^a^
	Complications**	3 (25%)	2 (9.5%)	-	-	-	0.328^a^
Mechanical ventilation		2 (16.7%)	2 (9.5%)	-	-	-	0.61^a^
Hospital care		2 (16.7%)	3 (14.3%)	-	-	-	1^a^
Mean hospital length of stay, days (SD)		14 ± 11.3	12 ± 9.5	-	-	-	0.853^f^
PICU		1 (8.3%)	2 (9.5%)	-	-	-	1^a^
Mean PICU duration, days (SD)		15	12 (9, 15)	-	-	-	-
**Microbiological variables**							
PCR *Bordetella pertussis* (positive)		12 (100%)	-	-	-	-	-
Median *Bordetella pertussis* load, log_10_ copies/ml (IQR)		6.1 (5.8–7.7)	-	-	-	-	-
Other pathogens grown in culture ***		0 (0%)	1 (4.8%)	-	-	-	1^a^
Respiratory Viruses (positive)	**Overall**	**8 (66.7%)**	**18 (85.7%)**	**2 (22.2%)**	**0.004** ^a^	**0.005** ^**b**^	0.377^a^
	Bocavirus	0 (0%)	1 (5%)^2^	0 (0%)	1^a^	1^a^	1^a^
	Flu	0 (0%)	1 (4.8%)	0 (0%)	1^a^	1^a^	1^a^
	Rhinovirus/Enterovirus	4 (33.3%)	13 (62%)	2 (22.2%)	0.116^a^	0.235^b^	0.223^b^
	Metapneumovirus	2 (16.7%)	2 (10%)^2^	0 (0%)	0.663^a^	0.559^a^	0.619^a^
	Coronavirus	2 (16.7%)	1 (4.8%)	0 (0%)	0.418^a^	1^a^	0.538^a^
	Adenovirus	0 (0%)	2 (10%)^2^	0 (0%)	0.707^a^	1^a^	0.529^a^
	Parainfluenzavirus	1 (8.3%)	6 (30%)^2^	0 (0%)	0.138^a^	0.299^b^	0.320^b^
	Respiratory syncital virus	0 (0%)	1 (4.8%)	0 (0%)	1^a^	1^a^	1^a^

Values expressed as No. (%), unless otherwise stated.

*Underlying conditions included respiratory disease (n = 1) and other (n = 1) for confirmed cases, cardiovascular disease for the possible case (n = 1) and lactose intolerance for the healthy control (n = 1).

** Complications included others (n = 3) in the confirmed cases, and Pneumonia-Encephalopathy (n = 1) and eating difficulty (n = 1) in the possible cases.

*** Another pathogen grown in culture was Moraxella catarrhalis.

^1^at least one dose.

^2^Data available for only 20 subjects.

^3^Data available for only 11 subjects.

^a^Fisher exact test.

^b^Chi-square test.

^c^Kruskal Wallis test.

^d^ANOVA test.

^e^Wilcoxon Rank Sum test.

^f^T-Student test.

Abbreviations: IQR, Interquartile Range; SD, Standard Deviation; PICU, Pediatric Intensive Care Unit.

### Molecular detection of *Bordetella* and respiratory viruses

Nasopharyngeal aspirates were collected from all patients included in the study. Bacterial DNA extraction was performed by Nuclisens® EasyMag® (bioMérieux, Marcy-l’Etolie, France) according to manufacturer instructions and concentrating 400 μL of specimen into 50 μL extracts for *Bordetella* real-time PCR and into 25 μL for further 16S rRNA gene sequencing. Viral DNA/RNA extraction was performed by MagnaPure (Roche Diagnostics, Paris, France) following manufacturer’s instruction. The quality and concentration of the resulting DNA/RNA was measured with Nanodrop (Thermo Fisher Scientific, Massachusetts, USA). Microbiological confirmation of *Bordetella* infection by real-time PCR was run on 5 μl of non-diluted extracts and was based on multiplex detection of the targets IS*481* (present in *B*. *pertussis*, *B*. *holmesii*, and some *B*. *bronchiseptica* isolates), pIS*1001* (*B*. *parapertussis*-specific) and the human internal control *rnaseP* gene. Two additional singleplex reactions confirmed the *Bordetella* species in IS*481*-positive specimens, by either the *ptxA*-Pr (*B*. *pertussis*-specific) or the hIS*1001* (*B*. *holmesii*-specific) gene targets [14]. *Bordetella* DNA load was calculated using the IS*481* gene [20]. In addition, the presence of viral respiratory infection in nasopharyngeal samples was tested by Allplex™ Respiratory Panels Assays 1, 2 and 3 (Seegene Inc., Seoul, Korea) targeting 16 viruses (Bocavirus, Influenza virus types A and B, Rhinovirus, Enterovirus, Metapneumovirus, Coronavirus types OC43, 229E and NL63, Adenovirus, Parainfluenza virus types 1, 2, 3 and 4, and Respiratory syncytial virus types A and B).

### 16S rRNA sequencing

The 16S rRNA gene amplicons were produced by using the degenerate universal bacterial primers 41F (5’- CCTACGGGNGGCWGCAG-3’) and 805R (5’- GACTACHVGGGTATCTAATCC-3’), targeting the variable regions 3 to 4 (V3-V4) (550 bp), according to the following cycling conditions: 2 min at 94°C, followed by 30 cycles of 10s at 94°C, 30s at 52°C, 90s at 68°C, and a final step of 7 minutes at 68°C. The PCR products were purified using a Minielute PCR purification Kit (Qiagen, Venlo, The Netherlands), followed by Agencourt AMPure beads (Beckman Coulter, Munich, Germany). NGS sequencing was performed at Instituto de Medicina Genómica, S.L. (IMEGEN, Valencia). NGS libraries were build using 12.5 ng of total DNA for each sample (previously concentrated at 5 ng/μl) using Nextera DNA Library Preparation kit (Illumina) according to the manufacturer’s instructions. DNA of negative controls (PBS) was extracted, amplified and sequenced by the same method implemented for the study samples.

### Bioinformatics analysis

Quality of sequencing data was assessed with FastX Toolkit (http://hannonlab.cshl.edu/fastx_toolkit/) which trimmed the low-quality ends from reads with a minimum Phred quality score set to Q10 after removing the first 16 base-pairs corresponding to the homologous sequences of the primers used. Amplicon sequence variants (ASVs) were binned and quantified using the DADA2 package [21] in R using the RDP set 16 taxonomic reference database [22]. Prior to decontamination, mean ± SD number of sequences per sample was 62220 ± 24888 and 442 ±166 per negative control. Decontam package [23] in R was used to remove potential environmental contamination. Moreover, reads that were not assigned to Kingdom Bacteria or were classified as such but no further taxonomic resolution was reached as well as reads that mapped against the human reference genome (hg38) were also excluded. Final mean number of reads per sample was 61661 [SD± 25174].

Alpha diversity metrics (Observed and Chao 1 for richness, and Shannon and Inverse Simpson indices for diversity) were calculated after collapsing reads at the genera level from rarefied samples at minimum read coverage per sample (9993 reads) using Phyloseq package [24] in R. For beta diversity analyses all reads were considered and collapsed at the genus level prior to calculating relative abundances using Phyloseq package [24].

To increase taxonomic assignment resolution for the ASVs identified as *Bordetella* genus, ASVs were blasted against the 16S rRNA gene reference database from NCBI. Best alignment hit was used for species taxonomic assignment.

A random forest classification model was built upon the entire bacterial genera dataset to evaluate the nasopharyngeal microbiota discriminatory power between confirmed and possible cases of whooping cough using the randomForest [25] R package with default parameters. The AUC-ROC was used as a measure of model performance. Direction of the association of each bacterial taxon to either confirmed or possible cases was estimated post-hoc with Cliff’s delta test.

### Statistical analysis

Categorical variables were examined with the Chi-square or Fisher-exact test (<25% of cells with <5 values) for description of the study population. Continuous variables were described as means and standard deviations (SD) or as median and interquartile range (IQR) values and were further analyzed using ANOVA (for normally distributed samples) or Kruskal-Wallis test (for non-normally distributed samples). Similarly, either for alpha diversity metrics or bacterial taxa relative abundance, differences between two groups were calculated with Wilcoxon Rank Sum test and for more than two groups using a Kruskal Wallis test with further pairwise Wilcoxon test.

## Results

### Study population

Nineteen pertussis confirmed cases less than 1 year old were attended at HSJD during the study period and 12 out of the 19 (63.2%) accepted to be included in the study (hereafter named *confirmed cases*). In addition, 21 patients with clinical criteria of whooping cough and negative real-time PCR for *Bordetella* (hereafter named *possible controls)* and 9 healthy controls were recruited and matched with confirmed cases. Hence a total of 42 children were included in this study. Median age of participants was 5 months [IQR: 2–7 months] and 66.7% of them were female. The three study groups were similar in age and gender. Clinical symptoms of whooping cough were also similar between confirmed and possible cases (Table 1). Proportion of infants with their mother vaccinated with *B*. *pertussis* was 41.7% among confirmed cases and 95.2% among possible cases (p-value = 0.0002). Proportion of viral respiratory infections was 86%, 67% and 22% among possible cases, confirmed cases and healthy controls, respectively (overall p-value = 0.004).

### Whooping cough was associated to lower bacterial genus richness and diversity compared to healthy controls

**Fig 1:**
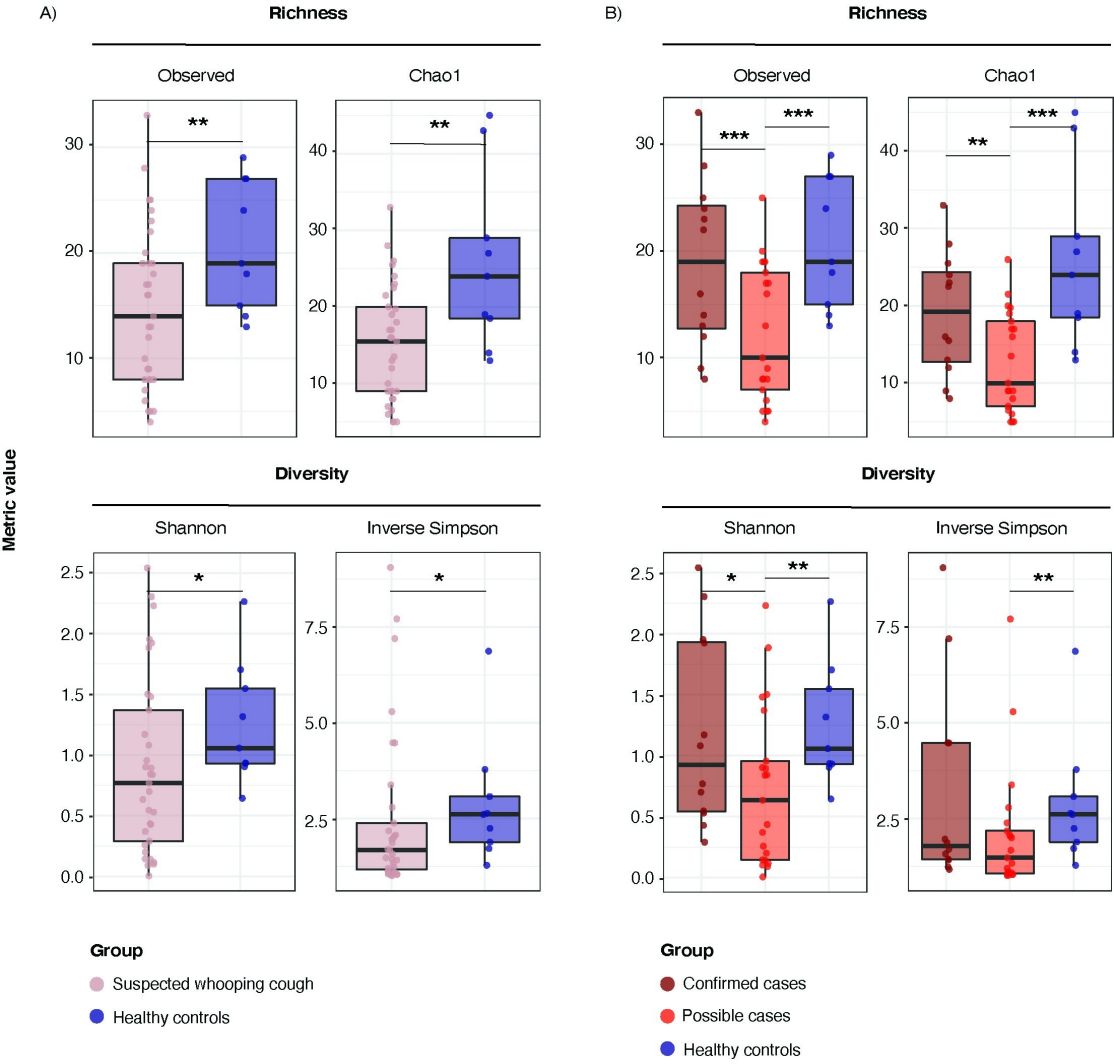
Alpha diversity metrics at the bacterial genus level between confirmed cases, possible cases and healthy controls. **A)** Boxplots showing the richness (Observed and Chao1 metric) and diversity (Shannon and Inverse Simpson indices) between the suspected whooping cough group (including confirmed and possible cases) and the healthy controls. **B)** Boxplots showing the richness and diversity between the confirmed cases, possible cases and the healthy controls. For both, A) and B), significance threshold is set as * p-value < 0.1, ** p-value < 0.05, *** p -value < 0.01.

Infants suspected of whooping cough, regardless of confirmatory *Bordetella* real-time PCR, presented significantly lower bacterial genus richness (Observed: 14 [IQR: 8–19] vs 19 [IQR: 15–27] / Chao1: 15.5 [IQR: 9–20] vs 24 [IQR: 18.5–29]) and trends for lower bacterial genus diversity than healthy controls (Shannon: 0.77 [IQR: 0.29–1.37] vs 1.06 [IQR: 0.93–1.55] / Inverse Simpson: 1.67 [IQR: 1.17–2.38] vs 2.6 [IQR: 1.88–3.07]) (Fig 1A). Among infants suspected of whooping cough, confirmed cases had significantly higher bacterial genus richness (Observed richness: 19 [IQR: 12.8–24.3] vs 10 [IQR: 7–18] / Chao1: 19.3 [IQR: 12.8–24.4] vs 10 [IQR: 7–18]) and trends for higher bacterial genus diversity (Shannon: 0.93 [IQR: 0.54–1.93] vs 0.63 [IQR: 1.45–0.95] / Inverse Simpson: 1.78 [IQR: 1.43–4.47] vs 1.47 [IQR: 1.07–2.18]) than possible cases (Fig 1B). Differences between confirmed and possible cases and healthy controls are also shown in Fig 1B.

### *Bordetella* was only found among the top ten most abundant bacterial genera in confirmed cases

**Fig 2:**
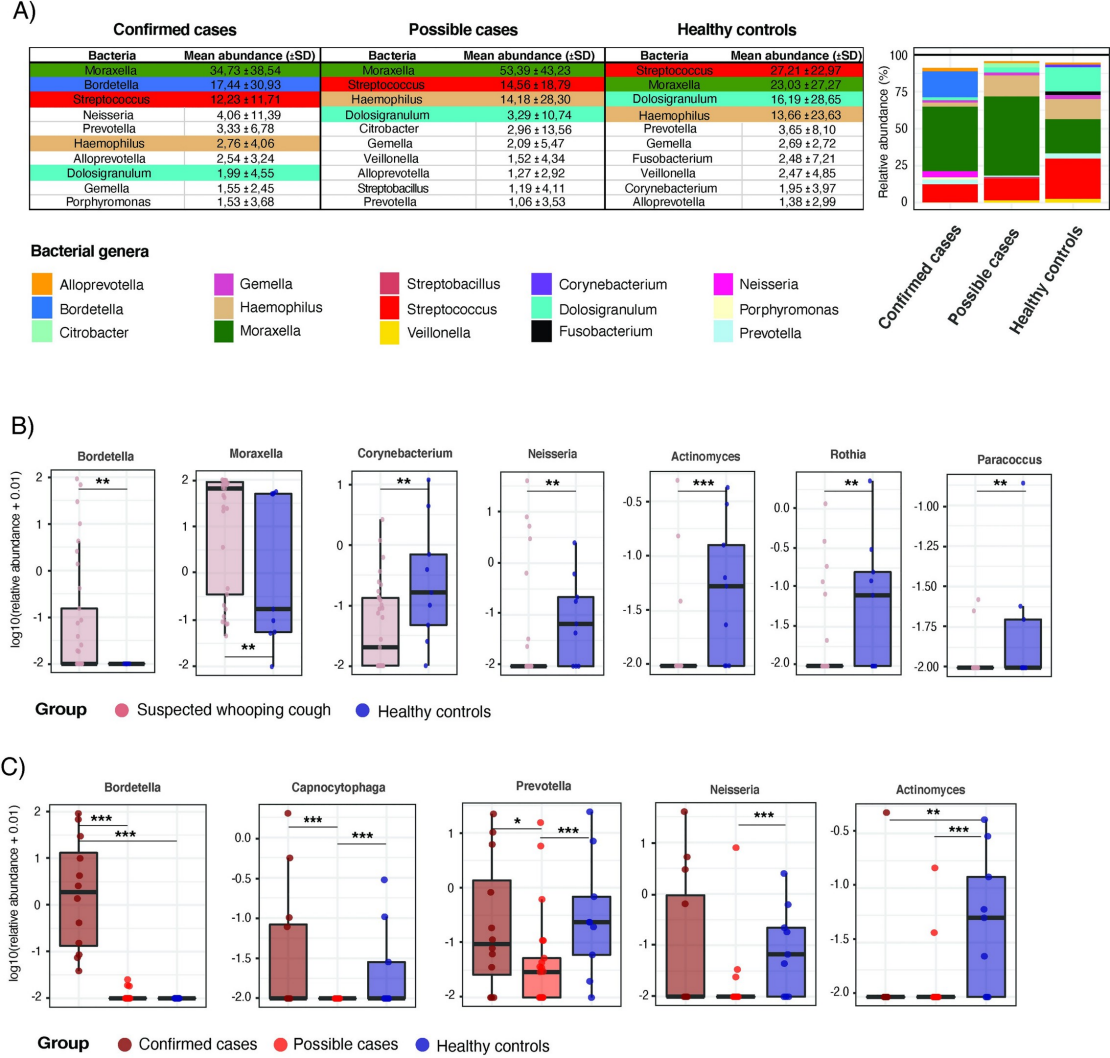
Nasopharyngeal microbiota bacterial genus composition profiling and differences between whooping cough children and healthy controls. **A)** Table showing the top ten abundant bacterial genera based on their mean relative abundance per group (mean ± SD). Color-code is kept through the groups to highlight the most abundant genera. On the right, barplots showing the mean contribution of the ten top bacterial genera per group in relation to the overall gut microbiota composition. **B)** Boxplots showing the relative abundance of those bacterial genera with a differential abundance between suspected whooping cough and healthy groups based on a Wilcoxon Rank Sum test (p-value significant threshold set to 0.05). Differences between groups are shown as * p-value < 0.1, ** p-value < 0.05, *** p -value < 0.01. **C)** Boxplots showing the relative abundance of those bacterial genera with an overall differential abundance between confirmed cases, possible cases and healthy groups based on a Kruskal-Wallis test (p-value significant threshold set to 0.05). Further unadjusted pairwise-statistical differences between groups are shown as * p-value < 0.1, ** p-value < 0.05, *** p -value < 0.01.

Mean relative abundance ranking of bacterial genera by group showed that *Bordetella* was only found within the top-ten in confirmed cases. Specifically, it was the second most abundant bacterial genus with a mean ± SD abundance of 17.44 ± 30.93 (Fig 2A). *Moraxella* and *Streptococcus* were consistently found among the top three most abundant genera in the nasopharynx in all three groups (Fig 2A). *Haemophilus* and *Dolosigranulum* were the third and fourth more abundant genera in healthy controls and possible cases while their mean abundance was lower in the confirmed cases (Fig 2A).

Compared to healthy controls, suspected whooping cough was associated to higher *Bordetella* and *Moraxella* as well as lower *Corynebacterium*, *Neisseria*, *Actinomyces*, *Rothia* and *Paracoccus* abundance (Fig 2B). Confirmed cases showed higher *Bordetella* abundance than both possible cases and healthy controls. Possible cases showed lower abundance of *Capnocytophaga* and *Prevotella* than both confirmed cases and healthy controls, and lower *Neisseria* abundance than healthy controls. Healthy controls showed higher *Actinomyces* abundance than both confirmed and possible cases (Fig 2C).

### *Bordetella* abundance by 16S rRNA gene sequencing was detected in all confirmed cases and 3 possible cases

**Fig 3:**
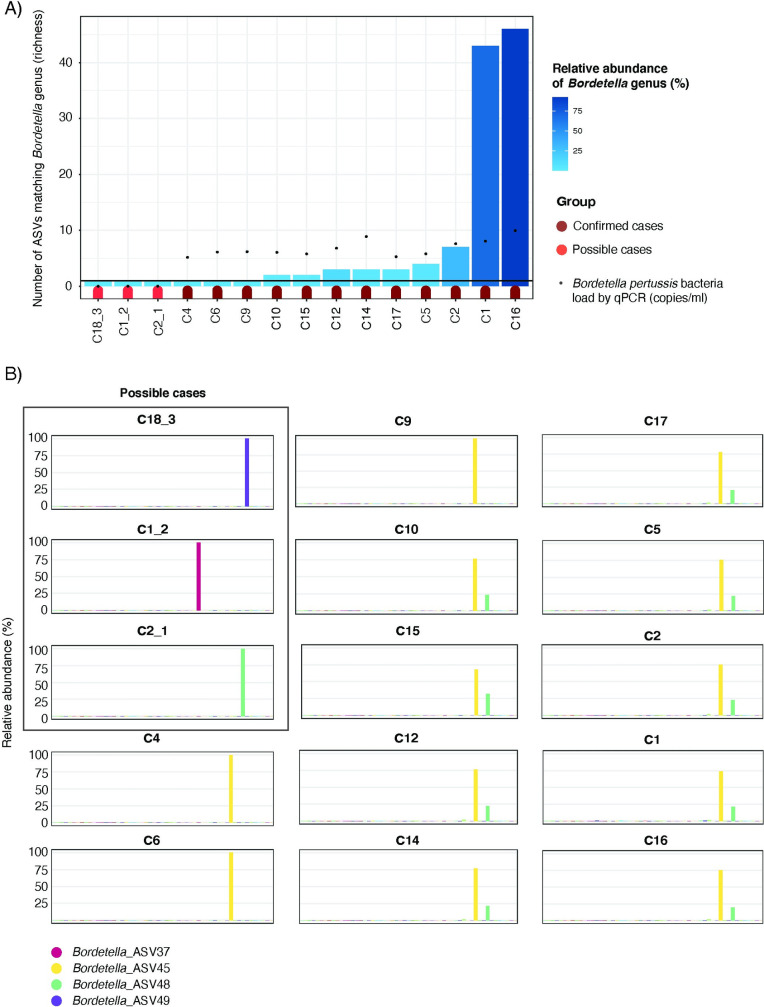
Increased *Bordetella* detection sensitivity by 16S rRNA gene sequencing. **A)** Barplot showing *Bordetella* detection by 16S rRNA gene sequencing. Height of bars represent the total number of unique 16S rRNA gene sequences (Amplicon Sequencing Variants, ASVs) matching *Bordetella* genus per sample. Red/magenta flags at the bottom of the bars represent possible and confirmed cases, respectively. Color-gradient in blue represents total relative abundance of *Bordetella* genus reads to overall bacterial composition per sample. Black dots show the *Bordetella* bacterial load detected by real-time PCR (copies/ml). **B)** Barplots showing the compositon of the overall *Bordetella* genus abundance at the single ASVs level. The x-axis represent each of the ASVs matching *Bordetella* genus (n = 55). The y-axis represent their contribution to overall *Bordetella* genus relative abundance (100%). Each ASV is colored differentently. More detailed color-coding for those ASVs with a major contribution is shown as the figure legend at the bottom.

Significant differences were observed according to *Bordetella* detection between 16S rRNA gene sequencing data and real-time PCR. *Bordetella* genus reads determined by 16S rRNA gene sequencing were found in all confirmed cases (12/12, 100%), 3 out of the 21 possible cases (14.3%) but not in healthy controls, hence increasing diagnosis sensitivity from 36.4% (12/33) to 45.5% (15/33) in suspected cases (Fig 3A). Overall, there was a good positive correlation between *Bordetella* bacterial load detected by real-time PCR and *Bordetella* ASVs’ richness (Spearman’s correlation R^2^ = 0.72, p-value = 0.0025) and *Bordetella* relative abundance (R^2^ = 0.87, p-value<0.001) detected by 16S rRNA gene sequencing.

Among the 55 *Bordetella* ASVs, a BLAST search identified 54 as *B*. *pertussis* (54/55, 98.2%) and 1 as *B*. *holmesii* (1/55, 1.8%). *Bordetella* genus abundance in all confirmed cases was dominated by either a unique ASVs (ASV45 in yellow) or by two ASVs (ASV45 in yellow and ASV48 in light green) (Fig 3B). Among the possible cases, *Bordetella* genus abundance was dominated by ASV37 in dark pink in subject C1_2, ASV49 in purple in subject C18_3 and ASV48 in light green in subject C2_1 (Fig 3B). After the BLAST search, ASV37 and ASV48 were both identified as *B*. *pertussis*, whereas ASV49 was identified as *B*. *holmesii*.

### Nasopharyngeal microbiota-based analysis had potential for accurate diagnosis of whooping cough

**Fig 4:**
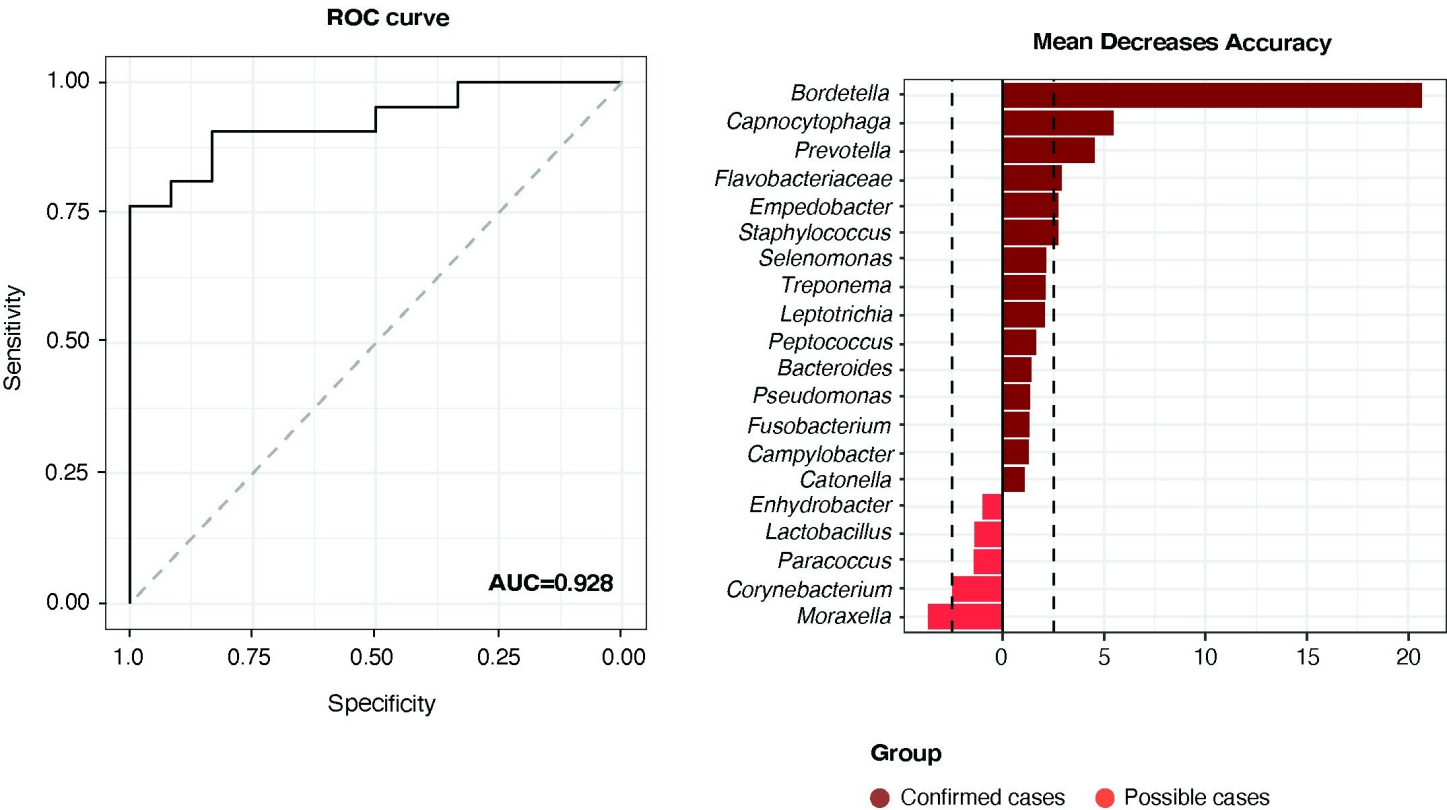
*Bordetella* genus is the best, although not unique, bacterial target for whooping cough diagnosis. **A)** The random forest model resulting AUC-ROC curve showing its performance to discern between confirmed and possible cases of whooping-cough based on the nasopharyngeal microbiota composition**. B)** Barplot showing the single variable analysis of the random forest model. The length of bars along the x-axis represents the Mean Decrease Accuracy (MDA) value for each bacterial taxon as a metric for its importance in the model. The MDA significance threshold is set to 1 and variables with an absolute MDA score >2.5 are highlighted. Bacterial taxa are ordered from more to less discriminative and split by the group they correlate to (red for possible cases, magenta por confirmed cases).

The resulting Area Under the Receiver Operating Characteristic curve (AUC-ROC) of the random forest model showed a good discriminatory power to discern between confirmed and possible cases (AUC = 0.928; 95% CI: 0.61, 0.91) (Fig 4A). At the single taxa level, *Bordetella* genus had the highest potential for classification with a mean decrease accuracy value up to 4 times higher any other taxa (Fig 4B). Other relevant taxa in the model included *Capnocytophaga* and *Prevotella* associated to confirmed cases, and *Moraxella* to possible cases.

## Discussion

Changes in the nasopharyngeal microbiota have been previously described in the context of respiratory diseases, but, to date, the composition of the whooping cough-associated microbiota and whether and to what extent it plays a role in disease outcome are unexplored. To the best of our knowledge, this study addresses for the first time the characterization of the nasopharyngeal microbiota composition in infants with possible and confirmed pertussis compared to healthy controls. In the present study we hypothesize that, on the one hand, confirmed whooping cough may not be related to profound changes on overall nasopharyngeal microbiota composition but to the appearance of *Bordetella* along with reduced abundance of beneficial bacteria. On the other hand, in possible cases, pertussis symptoms may not be directly related to *Bordetella* presence but to a less rich and diverse nasopharyngeal microbiota along with higher prevalence of viral coinfection.

Diverse longitudinal studies performed in newborns during the first year of life show how the nasopharyngeal microbiota changes over time and can either become a healthier or more aberrant community. In healthy infant populations similar in age to ours (5 months old), initial colonizers such as *Staphylococcus* and *Corynebacterium* are expected to be replaced by other more unstable genera including *Moraxella*, *Streptococcus* and *Haemophilus* [26, 27]. Our study identified *Moraxella* and *Streptococcus* as the major bacterial genera in the nasopharyngeal microbiota of infants regardless of whooping cough symptomatology. While these taxa were accompanied by *Bordetella* in the confirmed cases, *Haemophilus* and *Dolosigranulum* were found other major players in possible cases and healthy controls.

Bosch *et al*. reported higher rates of respiratory tract infections in children younger than 1 year old with an altered nasopharyngeal microbiota including decreased microbial community stability, prolonged decreases of *Corynebacterium* and *Dolosigranulum*, and an enrichment of *Moraxella* [28]. Moreover, *Moraxella* and *Haemophilus*, both members of the phylum Proteobacteria, have not only been related to asthma and bronchiolitis development, but also to frequent acquisition of respiratory viruses [29, 30]. In our study, the high presence of respiratory viruses in confirmed and possible cases agree with this finding. A recent study of our group found *Dolosigranulum* as a main component of the healthy nasopharyngeal microbiota in children as well as negatively associated to inflammatory states [31]. In the present study, children suspected of whooping cough showed higher *Moraxella* as well as lower *Corynebacterium* compared to healthy controls. Despite differences were not statistically significant, less *Dolosigranulum* was also found in the former. Healthy controls also showed higher abundance of *Actinomyces*. This finding agrees with results from Pettigrew *et al*. that reported *Actinomyces* being associated with better clinical course of pneumonia [32]. Hence, our results point towards reduced presence of beneficial bacteria and predominance of more unstable and potentially pathogenic bacteria in the context of pertussis. Our results also indicated that while the bacterial signature for confirmed cases seemed to be primarily driven by the presence of *Bordetella*, possible cases of whooping cough showed an interesting and more complex profile including lower alpha diversity, lower *Capnocytophaga*, *Prevotella* and *Neisseria* abundance, and higher viral co-infection rate than both confirmed cases and healthy controls. Despite there is no consistency in literature about whether respiratory disease states relate to lower or higher alpha diversity [33], this result is in line with that of a previous study linking lower Chao1 index to rhinovirus infection [34].

Interestingly, we found higher rates of respiratory viral co-infection among suspected whooping cough infants (confirmed and possible) compared to healthy controls, with the highest positivity in possible cases. *Ulrich et al*. reported in 2011 that viral co-infections were rare in *B*. *pertussis* infection [35], but our results are in line with those from Frassanito *et al*. that found viral co-infection in 47.2% of children younger than 6 months diagnosed of pertussis [36].

We included 33 children clinically suspected of whooping cough, but only 12 of them were confirmed by real-time PCR. Using 16S rRNA gene sequencing, diagnostic yield increased from 36.4% to 45.5% compared to real-time PCR. Our results are similar to those previously reported by Ding *et al*., who found the highest positive rate in whooping cough diagnosis by 16S rRNA gene sequencing data (66.7%) compared to either real-time PCR (59.8%) and microbiological culture (14.3%) [37]. While NAATs have significantly improved pertussis diagnostic sensitivity compared to the gold standard bacterial culture, there are still major caveats. On the one hand, the most common used target, the IS481 gene, is known to be present at high copy number (50–200 copies) in *B*. *pertussis*, but also present in different copy number in other non-pertussis species as *B*. *holmesii* and *B*. *bronchiseptica* [38]. Consequently, more sophisticated multiplex NAATs, including gene targets pIS1001, ptxA-Pr and hIS1001, used in this study, are required for proper identification of the different *Bordetella* species in clinical specimens. Moreover, a real-time PCR positive result is subjected to an arbitrary decision on the resulting Ct value and up to date there is some controversy on what Ct values >35 really mean [39, 40]. Since some findings suggest that other non-*pertussis* species can lead to whooping cough characteristic symptomatology [1] and we identified *B*. *holmesii* in one possible case, non-targeted 16S rRNA gene sequencing, may bring advantages not only in terms of sensitivity (any detected read assigned to *Bordetella* will be reported as positive) but also of specificity (any species will be detected), at least, in young children (<1 year old).

In addition to improve pertussis diagnostic, the benefit of running a non-targeted 16S rRNA gene sequencing analysis over directed NAATs is the amount of valuable information on all other bacterial species present that could be playing a role in the development of whooping cough symptomatology and could be useful for improved future therapeutic treatments. The main disadvantages of using a non-targeted microbiological diagnostic based on Illumina sequencing of a fraction of the 16S rRNA gene, are the long wet-lab procedures for library preparation and sequencing, as well as the limited taxonomic resolution reached at the species level. Both limitations could be overcome sequencing the entire 16S rRNA gene with portable sequencers such as MinION (Oxford Nanopore Technologies, UK)

Findings reported in this study are subject to limitations derived from the reduced sample size and the poor taxonomic resolution reached at the bacterial species level with the available taxonomic reference databases. However, we believe our results are representative since they include more than 60% of all whooping cough confirmed cases attended in the biggest pediatric Hospital in our country. In addition, while our targeted virome analysis included the major respiratory viruses known to infect and cause disease among children <1 year old, we are aware that a metagenomic sequencing approach would have covered a broader viral spectrum as well as other taxonomic groups such as fungi and other minor eukaryotes. Last, we were able to collect *B*. *pertussis* vaccination information for all children, but mother’s vaccination state could not be collected for the healthy controls. Nonetheless since 88.9% of the healthy children’s mothers were from Spain, it is plausible to assume they would have been vaccinated being the target group of the Spanish vaccination program for pregnant women [41].

In conclusion, *Bordetella* detection is the primary change on the nasopharyngeal microbiota of children with confirmed *pertussis*. Non-confirmed children with whooping cough symptomatology appear to have a differential nasopharyngeal microbiota composition with a potentially relevant role of respiratory viruses. NGS of the 16S rRNA gene has potential for improved whooping cough diagnosis in young children and opens a new avenue for microbiological diagnosis of other diseases with a suspected infections origin but with an undefined causative agent.
